# Preference for Averageness in Faces Does Not Generalize to Non-Human Primates

**DOI:** 10.3389/fnbeh.2017.00129

**Published:** 2017-07-11

**Authors:** Olivia B. Tomeo, Leslie G. Ungerleider, Ning Liu

**Affiliations:** Section on Neurocircuitry, Laboratory of Brain and Cognition, National Institute of Mental Health (NIMH), National Institutes of Health (NIH) Bethesda, MD, United States

**Keywords:** macaques, faces, averageness, visual preference, eye-tracking

## Abstract

Facial attractiveness is a long-standing topic of active study in both neuroscience and social science, motivated by its positive social consequences. Over the past few decades, it has been established that averageness is a major factor influencing judgments of facial attractiveness in humans. Non-human primates share similar social behaviors as well as neural mechanisms related to face processing with humans. However, it is unknown whether monkeys, like humans, also find particular faces attractive and, if so, which kind of facial traits they prefer. To address these questions, we investigated the effect of averageness on preferences for faces in monkeys. We tested three adult male rhesus macaques using a visual paired comparison (VPC) task, in which they viewed pairs of faces (both individual faces, or one individual face and one average face); viewing time was used as a measure of preference. We did find that monkeys looked longer at certain individual faces than others. However, unlike humans, monkeys did not prefer the average face over individual faces. In fact, the more the individual face differed from the average face, the longer the monkeys looked at it, indicating that the average face likely plays a role in face recognition rather than in judgments of facial attractiveness: in models of face recognition, the average face operates as the norm against which individual faces are compared and recognized. Taken together, our study suggests that the preference for averageness in faces does not generalize to non-human primates.

## Introduction

Faces provide a wealth of information for social communication and interaction in both humans and non-human primates. They convey information about an individual’s identity, its emotional state (e.g., happy), intention, focus of attention, etc. In humans, we also judge the attractiveness of an individual by his/her face. Most human studies assume that subjects make some common aesthetic/affective judgment for attractiveness (Rhodes, 2006). Facial attractiveness introduces multiple positive social consequences (e.g., eliciting positive personality attributions) and plays a critical role in certain social behaviors (e.g., mate assessment and the development of same-sex alliances; Rhodes, 2006). In general, opposite-sex judgments of facial attractiveness agree with same-sex ones due to the need for assessing same-sex danger as potential rivals for mates and/or generic aesthetic responses made to all faces (Langlois et al., 2000). Facial attractiveness is a long-standing topic of active study in both neuroscience and social science. Although research on facial attractiveness has been prolific over the past few decades, little is known at what point in our evolution facial characteristics became an important criterion to evaluate an individual’s attractiveness.

Non-human primates share similar social behaviors and underlying neural mechanisms with humans, especially for face processing. For example, like humans, non-human primates can read the emotional state (e.g., threat) from facial expressions (Kanazawa, 1996). Moreover, neuroimaging studies have found that the neural mechanisms underlying the perception of emotional facial expressions in monkeys are remarkably similar to those in humans (Hadj-Bouziane et al., 2008). Therefore, it is possible that non-human primates and humans share a common evolutionary basis for using facial traits. Then, one may ask whether non-human primates, like humans, also utilize facial traits to appraise the individual’s attractiveness and, more generally, which kinds of facial traits they prefer. Waitt and Little (2006) found that when facing a symmetrical and an asymmetrical version of the same face stimuli simultaneously, monkeys show visual preferences for the symmetric over asymmetric version, a finding aligned with results in humans (Rhodes et al., 1998; Jones et al., 2007). Moreover, female macaques exhibited preferences for red versions of male faces (Waitt et al., 2003). However, it should be noted that preferences for symmetry and coloration are robust biases in numerous visual domains (such as bodies) and throughout the animal kingdom (Brookes and Pomiankowski, 1994; Dixson, 1998; Domb and Pagel, 2001; Hughes et al., 2015). Therefore, to further our understanding of preferences for facial traits in non-human primates, here we considered a well-accepted and face-specific factor influencing attractiveness reported for humans, namely, averageness (Rhodes, 2006).

It has been long known that averageness is one of the major factors influencing judgments of facial attractiveness in humans (Galton, 1878). Adults rate average composite faces as more attractive than the individual faces used to construct them; the more faces that are blended together to create the average one, the more attractive the average face becomes (Langlois and Roggman, 1990; Little and Hancock, 2002). This signature is not altered by gender (between male and female faces/raters), though effect sizes may be even higher for same-sex ratings than opposite-sex ratings (Rhodes, 2006). Aside from average composite images, individual faces that are closer to the average face are considered to be more attractive than distinctive ones (O’Toole et al., 1994; Rhodes and Tremewan, 1996).

In adult human subjects, facial attractiveness is usually measured by self-reporting. Since this would be impossible to do with non-human primates as well as human infants, an alternative approach, preferential looking behavior, has been widely conducted in these subjects to establish visual preferences. This paradigm measures the spontaneous response to view stimuli attracting the interest of the viewer over other stimuli. Although cautious interpretation (e.g., interest vs. pleasure) of the preferential looking behavior is necessary, there is considerable evidence from human infants and adults that such behavior is positively linked to stimulus attractiveness. Indeed, infants look longer at faces rated as attractive by adults when presented in pairs with unattractive faces (Langlois et al., 1987; Slater et al., 1998). Adults also exhibit preferential looking towards faces that they judge to be more attractive (Hildebrant and Fitzgerald, 1978; Power et al., 1982). In non-human primates, analyzing looking behavior has also been utilized to establish visual preferences, such as preferences for conspecifics, symmetry and coloration (Fujita, 1987; Waitt et al., 2003; Waitt and Little, 2006). For example, macaques prefer observing pictures of the same species over those of the other species (Fujita, 1987). Therefore, in the present study, we analyzed looking behavior to determine whether monkeys show visual preferences for average monkey faces.

## Materials and Methods

### Participants

Three adult male rhesus macaques (monkeys B, I and S; *Macaca mulatta*; 9–11 years old) participated in this study. They were acquired from the same primate breeding facility in the United States where they had social-group histories as well as group-housing experience until their transfer to the National Institute of Mental Health (NIMH) for quarantine at the age of approximately 4 years. After that, they were individually caged with auditory and visual contact with other conspecifics in the same colony room, which accommodates about 20 rhesus monkeys. All subjects used in this study had been housed at NIMH for at least 5 years before this experiment. Thus, all three subjects had extensive social experience, which made them familiar with perception and interpretation of facial cues in conspecifics. Moreover, all subjects had similar prior experience with fixation as well as match-to-sample tasks.

All procedures followed the Institute of Laboratory Animal Research (part of the National Research Council of the National Academy of Sciences) guidelines and were approved by the NIMH Animal Care and Use Committee. Each monkey was surgically implanted with a headpost under sterile conditions using isoflurane anesthesia. After recovery, subjects were trained to sit in a plastic restraint chair with their heads fixed and face a computer monitor, on which visual stimuli were presented.

### Stimuli

Color photographs of 18 male adult rhesus macaques in full-face frontal views with neutral expressions and eyes looking forward were selected. The composite average face was created by averaging the 18 individual faces based on 68 key feature points located at the same feature on each individual face using Abrosoft FantaMorph 5 Deluxe software (^1^version 5.3.6). Though the averaging process might make the composite face more symmetrical than the individual faces from which it is derived, the increased symmetry does not fully account for the attractiveness ratings that humans make for the averaged face. The average face is representative of the mean tendency of a population, which makes it more attractive than individual faces used to construct it (Rhodes et al., 1999; Komori et al., 2009). In order to parse out the effect of averageness from the effect of symmetry on facial preferences, all individual faces and the average face were made perfectly symmetrical to eliminate the effects of symmetry on facial preferences. The images were then cropped with an identical, symmetrical outline so that external features, such as ears and jagged fur outlines, were eliminated to prevent distinct features from biasing viewing behaviors. Because the averaging process also smoothed the face texture, a Gaussian blur filter (at least 2-pixel radius based on the blur level, see below) was applied to all individual faces to reduce differences in quality between them and the average face. Finally, each individual face was color-matched to the average one using MATLAB customized scripts to equate the distribution of intensity and color. All stimuli were presented on a gray background. When viewed from approximately 57 cm (the distance between the monitor and the subject), images of faces subtended a visual angle of approximately 10° × 10°.

### Procedure

**Figure 1:**
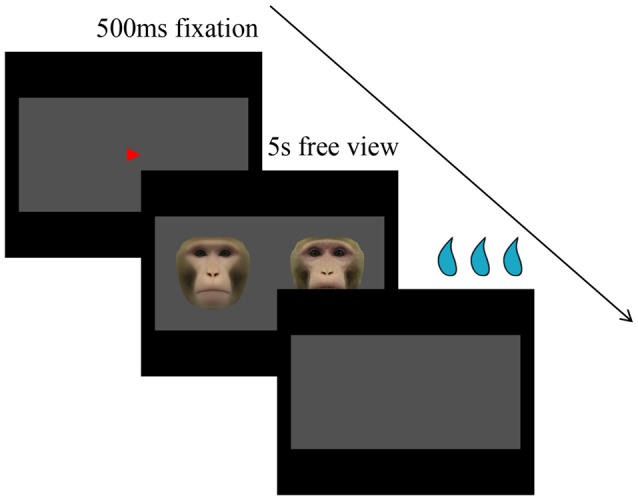
Visual paired comparison (VPC) task. Within each trial, after the monkey has fixated on the fixation spot for 500 ms, the fixation spot is extinguished, and a pair of faces appear and remain on the screen for up to 5 s, during which subjects are allowed to look at both images freely within the gray rectangle. Finally, the faces disappear and subjects are rewarded with juice.

Subjects were trained to perform a free-viewing visual paired-comparison (VPC) task. Eye positions were monitored by an infrared pupil tracking system (ISCAN, Inc., Woburn, MA, USA) with a 4-ms sampling rate. Each trial consisted of three periods: fixation, free viewing, and reward after correct responses or time out after errors. Each trial began with a fixation spot (red triangle, 0.4°) appearing in the center of a uniform gray rectangle 16° (H) × 32° (W; free-viewing window) against a black screen background. After the monkey had fixated on this spot for 500 ms, the fixation spot was extinguished, and a pair of faces (at ±8°) appeared and remained on the screen for up to 5 s, during which subjects were allowed to look at both images freely within the gray rectangle (Figure 1). Finally, the faces disappeared and subjects were rewarded with juice. If the monkey failed to maintain fixation long enough on the fixation spot or saccadic eye movements were made outside the free-viewing window for more than 500 ms, the trial was aborted without reward, which would be repeated later if the viewing time was less than 500 ms, and a new trial began after the 4-s time out. There was a 500-ms intertrial interval.

The experiment began with a training phase. Subjects were trained to perform the task with a set of non-face objects. During the test phase, each individual face was paired with one of the other 17 individual faces or with the average one. All pairs were presented four times with balanced locations (left or right side) for a total of 684 (19 × 18 × 2) trials per session. Two sessions were conducted for each subject. To control for novelty effects, the order of trials was pseudo-randomized so that each face was presented once every 9.5 trials.

### Data Analyses

Scan paths were defined as digitized eye movements calibrated in degrees of visual angle and superimposed on stimuli. For each subject, the total looking time (TLT) was measured for two rectangular areas of interest (AOIs) around each of the two faces shown on every trial. For each subject, the TLT was then averaged across trials for each pair to get the mean TLT for each of the two faces shown on such pairs. Trials in which subjects did not look at any of the two faces were excluded from the analysis.

To study whether macaques look at the average face differentially from individual faces when presented in pairs, we conducted a one-way repeated measures analysis of variance (ANOVA) based on pairs containing the average face. The pair factor in this ANOVA had two levels: average face and non-average face (individual face). The TLTs for both images in each pair were normalized (Equation 1) to control for variability among the subjects and then subjected to the ANOVA. We were also interested in testing whether there were any individual faces that macaques look at differentially from the other faces when presented in pairs. Accordingly, we did a one-way repeated measures ANOVA for each individual face.

(1) $${\text{\%TLT}}_{\text{A/B}}={\text{TLT}}_{\text{A/B}}/({\text{TLT}}_{\text{A/B}}+{\text{TLT}}_{\text{B/A}})$$

where TLT_A/B_ is the TLT for face A and TLT_B/A_ is the TLT for face B when face A is paired with face B.

We also investigated other factors that may have influenced judgments of facial attractiveness according to previous human work: blur level, contrast level and age of each face. The blur level of one image was computed via a no-reference blur metric that is in good agreement with observer ratings of subjective blur perception (Crété-Roffet et al., 2007). Blurrier images are represented by higher numerical values. The global contrast level of each image was computed via the root mean square (RMS) contrast, defined as the standard deviation of pixel intensities. Higher contrast images are represented by higher numerical values. The age of the average face was computed as the mean age of all the individual faces.

To better understand the attractiveness of individual faces, we also investigated the relationship between attractiveness of individual faces and their similarities to the average one. Previous studies have found that the psychophysical dissimilarity of complex objects (e.g., faces) can be represented by image-based measures conducted by the HMAX (Hierarchical Model and X) model (Yue et al., 2012), which is a popular computational model of neurobiologically plausible visual recognition (Riesenhuber and Poggio, 1999; Serre et al., 2007). Thus, in the present study, we scaled the dissimilarity (distance) of the individual face from the average one according to C2 units of HMAX model, which represents later stages of object processing in the ventral visual stream (inferior temporal cortex). We used the HMAX model implementation provided by http://cbcl.mit.edu/jmutch/cns/ (specifically, the HMAX package within the CNS simulation software). We trained the HMAX model on a set of monkey faces, so that C2 units would be face-selective. The Euclidean distances between C2 measures for each individual face and the average face were calculated. Individual faces that are more dissimilar to the average face are represented by longer distances.

Differences of these factors (i.e., blur, contrast, age and distance from the average face) between the two faces in each pair were treated as factors of covariance in the ANOVA analyses (Equation 2).

(2) $${\text{D}}_{\text{A/B}}={\text{F}}_{\text{A}}-{\text{F}}_{\text{B}}$$

where F_A_ is the factor level of face A and F_B_ is the factor level of face B.

We also included the subject as the nuisance factor in the ANOVA analyses. This procedure allowed us to test for above-mentioned factors of interest while also statistically controlling for variability among the subjects.

To further understand the factors that may have affected the TLT for faces, we analyzed relationships between each of our experimental factors (i.e., blur, contrast, age and distance from the average face) and the percent TLT. We calculated the difference in percent TLTs between the two faces in each pair (%TLT_A/B_ − %TLT_B/A_). Then, we computed partial correlation coefficients between the difference of percent TLT and the difference of each factor (e.g., distance from the average face), while controlling for effects of the rest of the factors (e.g., blur, contrast and age).

**Figure 2:**
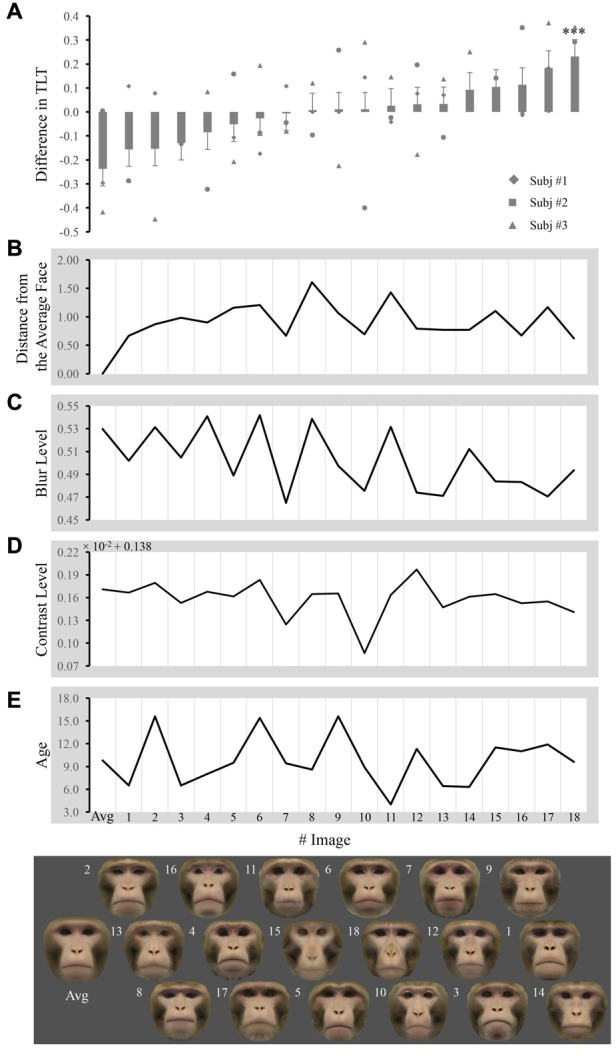
Difference in the total looking time (TLT) and obtained measures across faces. **(A)** Each bar represents the mean difference in the normalized TLT between the face labeled below the bar and the other 18 faces when paired together (unadjusted values for any covariates). The significance of the difference is given above/below the bars (adjusted *p* value for all the covariates, ***p < 0.001). Markers represent the mean difference from each subject. Each face image is placed exactly below the bar that reflects its TLT difference from others, so that the faces are ordered from left to right. The number next to image labels the individual number of faces. The distance from the average face, blur level, contrast level and age are plotted in **(B–E)** respectively for every corresponding image.

Note that the values shown in Figure 2A depict the raw, unadjusted values for any covariates. We did measure number of fixations and latency to look at each face but did not find significant results (data not shown).

## Results

The TLT for each face within one pair was averaged across trials for that pair and then normalized by the total TLT for both images in that pair in each subject. Differences between normalized TLTs of each image and the 18 other images when they were paired together are shown in Figure 2A. The distance from the average face, blur, contrast and age for each image are shown in Figures 2B–E, respectively.

We performed repeated ANOVAs with one factor pair to access whether macaques look at a certain face (e.g., the average face) differentially from the other faces when presented in pairs. For the average face, we found that the main effect of pair was not significant ([*F*_(1,47)_ = 0.395; *p* = 0.533]), indicating that macaques did not look at the average face differentially from individual faces when presented in pairs. We did find subjects looked longer at face #14 when paired with the other 18 faces (uncorrected *p* < 0.001, Bonferroni corrected *p* = 0.015).

**Figure 3:**
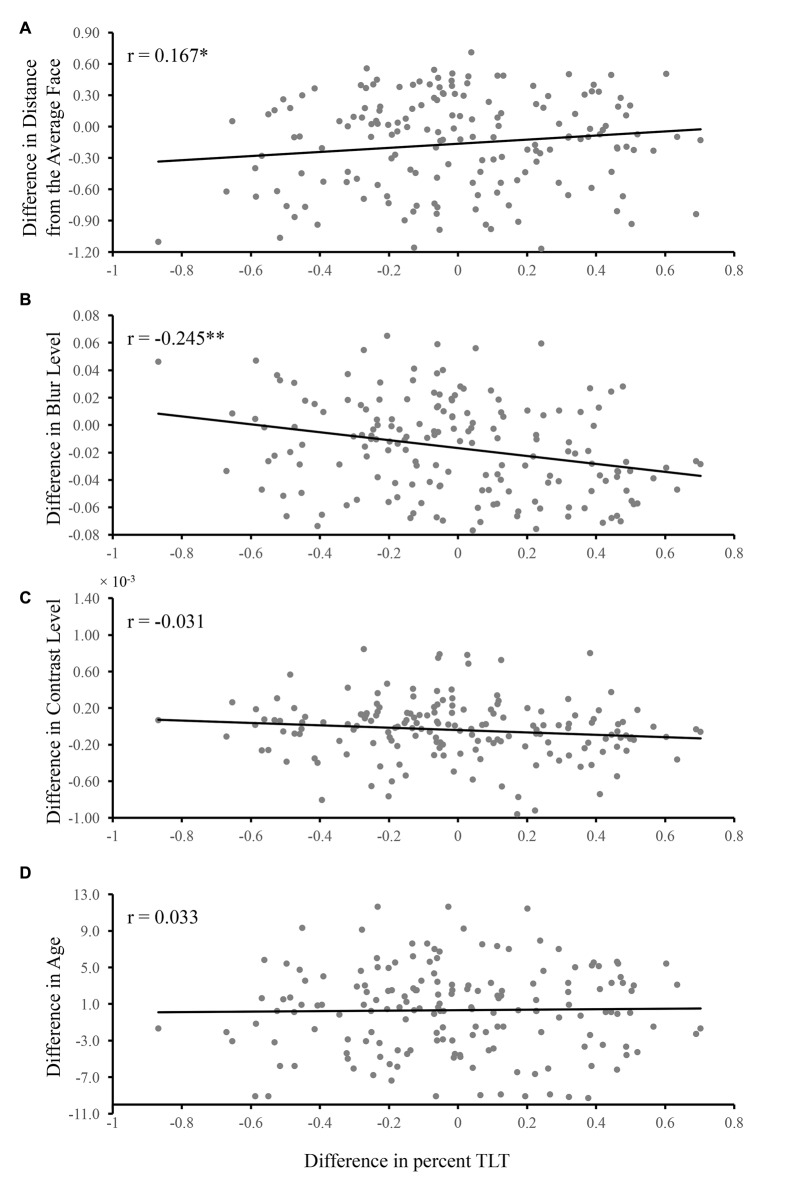
The relationship between the TLT and distance from the average face, blur level, contrast level and age. The difference of TLT, distance from the average face, blur level, contrast and age between the two faces in each pair were calculated based on formula 2 and 1. The partial correlations were conducted **(A)** between the difference of TLT and the difference of distance from the average face while controlling for the effects for the rest of the factors (i.e., blur, contrast and age); **(B)** between the difference of TLT and the difference of blur while controlling for the effects for the rest of the factors (i.e., distance from the average face, contrast and age); **(C)** between the difference of TLT and the difference of contrast while controlling for the effects for the rest of the factors (i.e., distance from the average face, blur and age); **(D)** between the difference of TLT and the difference of age while controlling for the effects for the rest of the factors (i.e., distance from the average face, blur and contrast). The correlation coefficients are given in each panel (*p < 0.05, **p < 0.01).

The partial correlation analysis revealed that there were correlations between the difference of percent TLT and the difference of distance from the average face (*r* = 0.167, *p* = 0.030, Figure 3A) as well as the difference of blur (*r* = −0.245, *p* = 0.001, Figure 3B), indicating that the larger the difference of distance from the average face/blur, the larger the difference of percent TLT between the two faces in each pair. The sign of the correlations indicated that the less blurred the face, the longer the looking time and the more dissimilar from the average face, the longer the looking time. No significant partial correlations were found between the difference of percent TLT and the difference of contrast (*r* = −0.031, *p* = 0.695, Figure 3C) or the difference of age (*r* = 0.033, *p* = 0.675, Figure 3D).

## Discussion

In the present study, we found that monkeys, unlike humans, showed no visual preference for the average face; the more the individual faces were dissimilar to the average face, the longer was the looking time. However, subjects did look longer at certain individual faces than others (including the average face). Moreover, there was a negative relationship between the visual preference for faces and blurring of faces. Below, we discuss the significance of these findings for understanding preferences for facial traits in macaques.

### No Preference for the Average Face

In the present study, we found that unlike humans (Rhodes, 2006; Little et al., 2011), rhesus macaques did not have the same visual preference for the average face. Other determinants of looking preferences (e.g., novelty and social position; Hauser et al., 1996) except for attractiveness, may not explain the current findings. In the present study, face images were taken from individuals located in several colony rooms (same as or different from the one where subjects were located). Subjects tested here had never/rarely seen 15 of the 18 individual faces and thus knew nothing/little about these individuals’ social positions. We did not find preferences/non-preferences for unfamiliar individual faces over familiar ones or within familiar ones. Moreover, the order of trials was pseudo-randomized to control for any novelty effects. Furthermore, the individual faces were not created by exaggerating any differences from average configurations (Rhodes et al., 2002), and did look ordinary. Therefore, in the present study, preferential looking found here may reflect attractiveness to some degree, though other factors may also be at play.

Although the preference for average faces has been widely confirmed in adults, it is weaker in children as compared with adults: the younger the age is, the weaker the preference is (Vingilis-Jaremko and Maurer, 2013), or even not present (i.e., in infants; Rhodes et al., 2002; Griffey and Little, 2014). These findings indicate that the preference for average faces must be at least partially learned from the surrounding culture. In the present study, we did find that all the three subjects, who have been housed at the same facility for more than 5 years, looked at certain individual faces statistically longer than others. That is, monkeys may learn the preference for faces from the surrounding social environment as humans. Therefore, the inconsistent results between monkeys and humans in the preference for average faces may be attributed to differences in the social environment between these two species.

Accumulated evidence has suggested that preferences for attractive faces in humans may be part of our biological heritage in addition to the social heritage (Rhodes, 2006; Little et al., 2011). In humans, facial attractiveness affects mate choice and then evolve through sexual selection. Then, preferences for certain facial traits may be a key characteristic that is passed generationally to be preserved throughout evolutionary history. These characteristics may also be utilized to assess same-sex danger as potential rivals for mates or develop same-sex alliances since same-sex judgments of facial attractiveness usually agree with opposite-sex ratings (Langlois et al., 2000). Previous studies have found that monkeys attract mates by secondary sex characteristics, such as body size and skin coloration of the anogenital region, which is used in sexual displays (Dixson, 1998; Domb and Pagel, 2001; Waitt et al., 2003; Hughes et al., 2015). Though only same-sex preferences for faces were tested here, our results provide new evidence to support such a view that faces may play a different role in certain social behaviors (e.g., same-sex danger and/or mate assessment) in monkeys and humans.

Contrary to findings in humans, we found that monkeys did not prefer to look at the average face. Moreover, the more the individual faces were similar to the average face, the shorter the looking time was. These findings are consistent with an earlier electrophysiological study, which showed that face-responsive cells in the rhesus macaque inferior temporal cortex are most often tuned around the average face, with increased response amplitude as the faces get farther away from the average face (Leopold et al., 2006). The consistency between the current behavior and previous physiological data suggests that the average face may act as a baseline against which other faces are compared for the purpose of discriminating among the many different faces one observes. That is, our data support the norm-based coding model of face recognition (Valentine, 1991; Valentine et al., 2016) and indicate that the average face likely plays an important role in recognizing individual faces rather than in judgments of attractiveness in macaques.

### Other Factors Influencing Visual Preferences

In the present study, we also investigated other factors that have been shown to influence judgments of facial attractiveness in humans: age, facial contrast and blurring. In humans, although youth influences facial attractiveness in both sexes, this component is more important for female facial attractiveness since it is a cue to reproductive value (Thornhill and Gangestad, 1999). Here, tested faces were male, which might account for the absence of age as a factor affecting face preferences. Previous studies in humans indicate that facial contrast influences judgments of facial attractiveness by impacting perceptions of youth in humans: faces with greater facial contrast look younger and more attractive (Porcheron et al., 2013). Here too, we did not find any influence of facial contrast on visual preferences in monkeys.

One factor that did affect visual preferences for faces in the present study was the level of blur: monkeys preferred less blurred faces over more blurred ones, an opposite effect to the one reported in humans (Little and Hancock, 2002; Jones et al., 2015). In humans, smooth or unblemished skin texture indicates youth, which is usually preferred, as mentioned above. However, since monkey faces are covered with fur, the smoothed (blurred) skin texture may appear to be less realistic. It has been shown that monkeys prefer viewing real conspecific faces rather than unrealistic cartoon faces (Yu et al., 2012). Therefore, different perceptions of blurring facial texture between monkeys and humans may account for the different findings between these two species.

In summary, our study provides new information about visual preferences for facial traits in monkeys: the average face may not influence judgments of attractiveness, but may be the norm to aid in face recognition. We believe these findings will be of interest for both human and monkey researchers who aim to understand the underlying neural mechanisms of face processing.

## Author Contributions

OBT and NL: study conception and design, acquisition of data, analysis and interpretation of data, drafting of manuscript. LGU: study conception and design, interpretation of data, drafting of manuscript.

## Conflict of Interest Statement

The authors declare that the research was conducted in the absence of any commercial or financial relationships that could be construed as a potential conflict of interest.

## Footnotes

^1^http://www.fantamorph.com
